# Comparative analysis of minimally invasive endoscopic versus conventional open posterior brucellosis lesion removal in the treatment of brucellosis spondylitis

**DOI:** 10.3389/fmed.2025.1573347

**Published:** 2025-07-04

**Authors:** Sikandaer Siyiti, Lin Hang, Adila Siyiti, Sui Jiangtao

**Affiliations:** ^1^Spinal Surgery, Sixth Afliated Hospital of Xinjiang Medical University, Xinjiang, China; ^2^The Sixth Clinical Medical College, Xinjiang Medical University, Xinjiang, China; ^3^Pathology Department, Affiliated Cancer Hospital of Xinjiang Medical University, Xinjiang, China

**Keywords:** brucellosis, brucellosis spondylitis, lesion removal, endoscopy, minimally invasive

## Abstract

**Objective:**

This study aims to evaluate the clinical effectiveness of minimally invasive versus conventional open posterior methods for the excision of brucellosis lesions in the context of spondylitis treatment. The findings are intended to inform and guide clinical practice.

**Methods:**

Forty-three patients with brucellosis spondylitis who attended our hospital from January 2020 to June 2023 were selected and divided into minimally invasive endoscopic brucellosis lesion removal (group A) *n* = 18 and traditional open lesion removal group (group B) *n* = 25 according to the operation type. All patients were given antibiotic treatment before operation. Analyze the relevant clinical indicators.

**Results:**

① There were no statistically significant differences (*p* > 0.05) between the two groups in terms of age, gender, body mass index (BMI), medical history, erythrocyte sedimentation rate (ESR), C-reactive protein (CRP), procalcitonin (PCT), D-dimer, hemoglobin (Hb), visual analog scale (VAS) score, Oswestry Disability Index (ODI) score and recurrence rate. ② The operation time (*p* < 0.012), intraoperative blood loss (*p* < 0.012), and postoperative hospital stay (*p* < 0.012) in group A were significantly shorter than those in group B, and the differences between the two groups were statistically significant (*p* < 0.05). No significant differences were observed in the remaining outcome measures.

**Conclusion:**

The results of this study showed that minimally invasive endoscopic brucellosis lesion removal could achieve the same efficacy as compared with traditional open posterior lesion removal, but minimally invasive surgery has the advantages of shorter operative time, lower intraoperative hemorrhage and more obvious advantages in postoperative rehabilitation, etc., which makes it clinically feasible and effective procedure.

## 1 Introduction

Brucellosis represents a zoonotic condition attributed to bacteria from the Brucella genus, posing health risks through several transmission routes. Humans can contract the disease by consuming unpasteurized animal products, including raw milk and dairy products such as soft cheeses, butter, and ice cream. Additional transmission pathways include skin or mucous membrane contact with these infectious agents, direct interaction with animals harboring the bacteria, or inhalation of aerosolized particles containing Brucella (1). The World Health Organization classifies brucellosis among the ‘seven neglected endemic zoonoses,’ noting its prevalence surged markedly during the recent pandemic of New Crown Pneumonia (2). The socio-economic impact of brucellosis is considerable, encompassing the extensive costs associated with medical treatment and the economic fallout from workdays lost due to illness (3). A notable manifestation of the disease, Brucellosis spondylitis (BS), primarily targets the spinal column. Brucella organisms predominantly infect the lumbar spine, although infection can also spread to the thoracic and cervical regions of the spine (4). The disease can present with acute symptoms including fever, severe headaches, joint and lower back pain, sometimes accompanied by diarrhea. Alternatively, the presentation can be more insidious, characterized by mild general discomfort, myalgia, and pains in the neck and back, often followed by nocturnal and intermittent fevers (5). Currently, the first choice for the treatment of Brucella spondylitis is pharmacological therapy (6). However, when pharmacological treatment is ineffective or ineffective, surgical treatment is the best option. Currently, the majority of patients presenting for medical evaluation are diagnosed at an advanced stage of the condition, where they typically report experiencing pain in the spinal area involved and neurological manifestations in the lower extremities. These symptoms often suggest the presence of inflammatory granulomas or abscess formations within the spinal canal or adjacent to the vertebrae. Such conditions are likely to exert direct or indirect pressure on the spinal cord, the cauda equina, or nerve roots. Given these circumstances, the necessity for prompt and effective surgical intervention becomes paramount to prevent further deterioration and ensure patient recovery (7).

The surgical approach to treating brucellosis spondylitis focuses on two critical aspects: This will be done firstly, to ensure complete removal of all infected lesions to prevent spread of infection in the spinal territory, secondly, to administer a regimented application of anti-infective treatment. The objective of this dual strategy is to both eradicate the infection and rehabilitate spinal function in order to reestablish spinal structural stability thereby improving patient outcome and quality of life. Typically, spinal infections are treated with traditional surgery in which large incisions are made in the patient’s spinal area and infected vertebral body and disk tissues are taken out to ensure complete treatment (8). While the traditional open procedure does have many advantages and is the procedure of most of the surgeons it is more important to let the surgeon decide for his patient which method is the best for his particular condition. Endoscopic debridement of brucellosis is a new departure of minimally invasive technique. In the procedure, doctors cut small incisions in the skin through which endoscopic techniques are used to reach the patient’s target vertebral region. The surgeon can then see the infected vertebrae and disks much more clearly via the endoscope and such procedures as lesion removal may be exercised (9).

In this study, we conducted a retrospective analysis by comparing 43 patients who were treated with minimally invasive endoscopic brucellosis lesion debridement and conventional open posterior lesion debridement for the treatment of brucellosis spondylitis, respectively, to investigate the differences between these two procedures in the treatment of brucellosis spondylitis.

## 2 Subjects and methods

### 2.1 Study design

This was a retrospective comparative evaluation.

### 2.2 Timing and location

The investigation was carried out between January 2020 and June 2023 at the Department of Spine Surgery, Sixth Affiliated Hospital of Xinjiang Medical University.

### 2.3 Study population

Analysis included clinical data from 43 patients who had been diagnosed with brucellosis spondylitis. These patients underwent surgical treatment either through minimally invasive endoscopic techniques or traditional open posterior lesion removal at the same facility during the study period. The cohort was divided into two groups, with 18 patients in the minimally invasive endoscopic lesion removal group (Group A) and 25 in the traditional open posterior lesion clearance group (Group B). Ethical approval for the study was granted by the Ethics Committee of the Sixth Affiliated Hospital of Xinjiang Medical University.

### 2.4 Inclusion criteria

The study included patients (i) diagnosed with brucellosis spondylitis based on clinical symptoms, laboratory findings, and imaging studies; (ii) who showed no improvement following antibiotic treatment; (iii) experiencing severe and persistent pain; (iv) with significant spinal neurological deficits; (v) who received either minimally invasive endoscopic or traditional open lesion removal surgeries; and (vi) who had a minimum follow-up duration of 6 months with complete data available.

### 2.5 Exclusion criteria

Excluded were patients (i) with target vertebrae affected by other spinal conditions in addition to brucellosis spondylitis, such as lumbar disk herniation, spinal fractures, or spinal tumors; (ii) with concurrent active infectious diseases like active tuberculosis or intestinal tuberculosis; (iii) presenting with lesions spanning three or more spinal segments; (iv) suffering from severe neurological or psychiatric disorders or other serious illnesses that could interfere with pain assessment; (v) with severe spinal deformities.

### 2.6 Diagnostic criteria for brucella spondylitis

(i) low back pain, fever, fatigue, weight loss, spinal lesion pain and percussion pain; (ii) X-ray early no special performance, a few weeks after the narrowing of the intervertebral space, the end plate above and below the bone density inconsistency; (iii) CT suggests that the vertebral body edges of the small and multiple destructive foci, foci of peripheral hyperplasia sclerosis, intervertebral disk destruction; (iv) MRI early hints of the involvement of the interstitial space above and below the vertebral body in the T1 image of the low signal, the T2 image of the high signal, the disk was uneven high signal; (v) C-reactive protein, erythrocyte sedimentation rate increased; (vi) standard test tube agglutination test results > 160:160. The intervertebral disks showed uneven high signal; (vii) C-reactive protein and erythrocyte sedimentation rate were elevated; (viii) Standard test tube agglutination test result was >1:160.

### 2.7 Surgical methods

#### 2.7.1 Preoperative preparation

After detailed history and physical examination, relevant imaging examinations (X-ray, CT, MRI and other auxiliary examinations) should be completed to assess the degree of severity of the patient’s illness. For patients presenting with concurrent health issues, specific management goals are critical. For those diagnosed with hypertension, stringent control of blood pressure is required, maintaining levels beneath 160/100 mmHg. Diabetes patients must adhere to strict glycemic controls, aiming for fasting blood glucose to be sustained below 8 mmol/L, while postprandial blood glucose 2 h after eating should not exceed 10 mmol/L. Additionally, it is imperative to manage urinary glucose to remain within the range of + to ++. The designated medical regimen before surgery involves administering doxycycline at a dosage of 100 mg orally twice daily, rifampicin 600 mg orally once a day, and gentamicin 320,000 units intravenously per day. This course of treatment should continue for at least 2 weeks. The timing for elective surgery is determined based on a marked improvement in the patient’s clinical symptoms and physical health, coupled with a significant reduction in inflammation markers, specifically when CRP is reduced to 20 mm/h or less and the ESR falls to 50 mm/h or lower.

#### 2.7.2 Surgical methods

(1) Open group: General anesthesia was given to all patients. Take patients with lumbar 3–4 brucellosis spondylitis as an example. After the general anesthesia took effect, the patient took the prone position, and the sterile towel sheet was routinely disinfected. Taking the lumbar 3–4 spinous process as the center, a posterior median incision with a length of about 8 cm was made, and the skin, subcutaneous tissue and fascial layer were incised sequentially, and the muscular layer was bluntly separated to the outer edge of the articular eminence with a bone cutter to stop the bleeding sufficiently, and the joints of the bilateral lumbar 3–4 vertebral plates and the joints of the joints of the bilateral lumbar 3–4 segments were exposed after the detection of no active bleeding. The bone knife was used to remove the bilateral articular synovial joints of lumbar 3–4, the hypertrophied ligamentum flavum was removed, and the spinal canal and nerve root canal were enlarged. In the course of the surgical exploration, the team encountered inflammatory adhesions surrounding the nerve roots. Utilizing specialized nerve root hooks, the surgeons meticulously retracted the dura mater and the nerve roots to expose the underlying structures. It was observed that the intervertebral disks between the lumbar 3–4 levels had sustained significant damage. The affected disk material was carefully excised using nucleus pulposus forceps designed for precision removal. Subsequently, the operation continued with the use of various tools including a reamer, a scraping spoon, and further use of nucleus pulposus forceps, all aimed at thoroughly removing any remaining diseased disk tissue and scraping away the endplates to prepare the site for healing. After ensuring adequate decompression of the nerve roots, a careful examination was conducted which confirmed the absence of residual compression or significant bleeding within the surgical site. The area was then subject to meticulous hemostasis to prevent any potential postoperative bleeding. The surgical team performed a thorough irrigation of the incision site with sterile saline to minimize infection risks. Each side of the surgical site was equipped with a negative pressure drainage tube to facilitate fluid evacuation. Finally, after a precise count of all surgical gauze and instruments, confirming that nothing was amiss, the incision was closed in layers using sutures, ensuring a secure and clean closure of the surgical wound.
(2) Minimally invasive group: General anesthesia was given to all patients. Take the patient with lumbar 4–5 brucellosis spondylitis as an example. During the surgical procedure, the radiofrequency knife was employed to meticulously peel away the soft tissues adhering to the surface of the vertebral plate. This step revealed the crucial junctions between the upper and lower articular synapse joints, as well as the upper edge of the vertebral plate and the ligamentum flavum in the lower vertebral body. The next phase involved precise surgical excision using a circular saw, bone cutter, and gun pliers. These tools facilitated the removal of the medial margins of the lower articular synapse joints at the lumbar 4 level and the medial margins of the upper articular synapse joints at the lumbar 5 level on the affected side. Subsequent to the resection of the ligamentum flavum, medullary forceps were utilized to meticulously clear the peripheral fat, effectively exposing the underlying nerve root. Upon exposure, it was evident that the disk exhibited signs of inflammation, with pus visibly exuding from it. The dead bone of lumbar 4–5 vertebral body was removed, and after sufficient decompression, the nerve root was no longer compressed, and there was no obvious bleeding, so an intervertebral drainage tube was left in place, and the wound was closed with layer-by-layer suture after counting the gauze and instruments without error.

#### 2.7.3 Post-operative management

1. To safeguard against infection, intravenous antibiotics were administered to patients 24 h following the surgical procedure, complemented by the prescription of non-steroidal anti-inflammatory drugs aimed at mitigating postoperative pain. 2. The surgical site’s drainage tube was scheduled for removal once the output recorded was less than 30 mL over a 24-h period, ensuring efficient management of wound fluids. 3. Postoperative protocol mandated patients to maintain bed rest, encouraging periodic movement of the lower limbs to circumvent the risk of deep vein thrombosis in the extremities. 4. A brace, to support the lumbar region was also provided, and was to be worn starting from the first to the third day post-surgery, for the region to assume the right spinal alignment and support. 5. At least 3 months’ continuation of the regular medication regimen used before surgery and consistent monitoring of liver and kidney functions to preclude the possibility of long term medication adverse effects were prescribed. 6. Diagnostic reviews based on X-ray and CT scans were performed, before the patient’s discharge, to ensure integrity of the bone grafting and accurate placement of the surgical hardware. 7. To assess the adequacy of decompression and thoroughness of the lesion removal in achieving the surgical goal, an MRI was conducted. 8. The optimal healing regimen was to provide lumbar support for t3 months, to continue this support through the critical healing phase to allow optimal healing of the spine.

### 2.8 Post-operative management

(1) VAS Score for Low Back Pain: This metric is employed to accurately gauge the intensity of a patient’s pain. The patient self-administers this assessment by marking a specific point along a 10 cm linear scale. This scale starts at 0, indicating no pain, and extends to 10, which represents the most extreme pain imaginable. The patient’s personal assessment helps provide a quantifiable measure of pain intensity.
(2) ODI for Low Back Pain: This index is pivotal in the management of spinal disorders, offering a nuanced view of patient progress in routine clinical practice. It is structured around 10 queries, focusing on daily living aspects such as pain severity, self-care proficiency, ability to lift, mobility in walking, capacity to sit and stand, quality of sleep, impact on sexual and social life, and ability to travel. Each question allows for six responses, ranked from 0 to 5, where choosing the first response scores zero, and selecting the final response garners the maximum of five points. The overall score is computed by dividing the actual score by 50, the highest possible total, and converting this result into a percentage. If a response is missing, the total possible score adjusts to 45, and the scoring method adapts accordingly.
(3) Measurement of Intraoperative Blood Loss: This calculation involves the aggregation of the postoperative weight of the blood-soaked gauzes plus the volume of blood collected in the suction device, subtracting any saline volume used for intraoperative rinsing. In specific terms, a small gauze totally saturated is about 30 milliliters of blood and a large gauze totally saturated is approximately 180 milliliters of blood loss. Using this exact method, the loss of blood can be estimated accurately at the time of surgery.

### 2.9 General information

(1) From January 2020 through June 2023, our institution conducted a detailed retrospective analysis of clinical data for 43 patients with brucellosis spondylitis. The patients herein were subjected to surgical interventions which included minimally invasive endoscopic debridement or traditional open posterior debridement of lesions due to brucellosis. The cohort was methodically divided based on the type of surgical procedure employed: Group A consisted of 18 patients who underwent minimally invasive endoscopic debridement and Group B totaled 25 patients who underwent traditional open posterior lesion debridement. Outcomes between two groups was extensively compared. Gender, age, body mass index and exhaustive previous medical histories were noted down with precision and analyzed in terms of any relationships with treatment outcome and recovery profile.

### 2.10 Observational indicators

Patient’s age, gender, body mass index, past history, duration of surgery, intraoperative bleeding, postoperative hospital stay, complications, pre- and post-operative VAS score, ODI score, ESR, CRP, PCT, D-dimer, Hb.

### 2.11 Statistical method

All statistical analyses were performed using SPSS version 26.0 (IBM Corp.). Continuous variables - including age, body mass index, operative time, intraoperative blood loss, length of postoperative hospitalization, VAS scores, ODI scores, ESR, CRP, PCT, D-dimer levels, and Hb concentrations - were assessed for normality using the Shapiro–Wilk test. Normally distributed variables were expressed as mean ± standard deviation and analyzed using independent samples t-tests, while non-normally distributed variables were presented as median (interquartile range) and analyzed using Mann–Whitney U tests. Categorical variables—including gender, medical history (cardiovascular disease, respiratory disease, endocrine disorders, metabolic diseases), and complications (dural tears, muscular venous thrombosis, deep vein thrombosis, surgical site infection, cerebrospinal fluid leakage, wound edge necrosis, sinus tract formation, and recurrence rates) - were expressed as counts (percentages) and analyzed using χ^2^ tests or Fisher’s exact tests, as appropriate. Preoperative and postoperative comparisons were made pairwise. To control confounding factors, multiple linear/logistic regression analysis was adopted, and covariates such as age and gender were included. For the problem of multiple comparisons, this study conducts the main hypothesis test. A conservative Bonferroni correction was adopted to adjust the significance threshold to 0.05/12 = 0.004. All *p* values were reported as both the original values and the corrected values. Among them, a corrected *p* value <0.004 was considered statistically significant. For exploratory analyses (such as laboratory indicators), the results are presented as uncorrected *p*-values, but their exploratory nature is clearly marked.

## 3 Results

### 3.1 Analysis of the number of participants

In this retrospective analysis, a total of 43 patients diagnosed with brucellosis spondylitis were treated either by minimally invasive endoscopic debridement or by traditional open posterior debridement of their brucellosis lesions. The cohort was stratified based on the type of surgical intervention they received: 25 patients were treated using the traditional open posterior approach, while 18 patients underwent the minimally invasive endoscopic approach. All patients from both groups were included in the outcome analysis, with a complete dataset available, ensuring no instances of data attrition were reported.

### 3.2 Test flow chart

**Figure 1:**
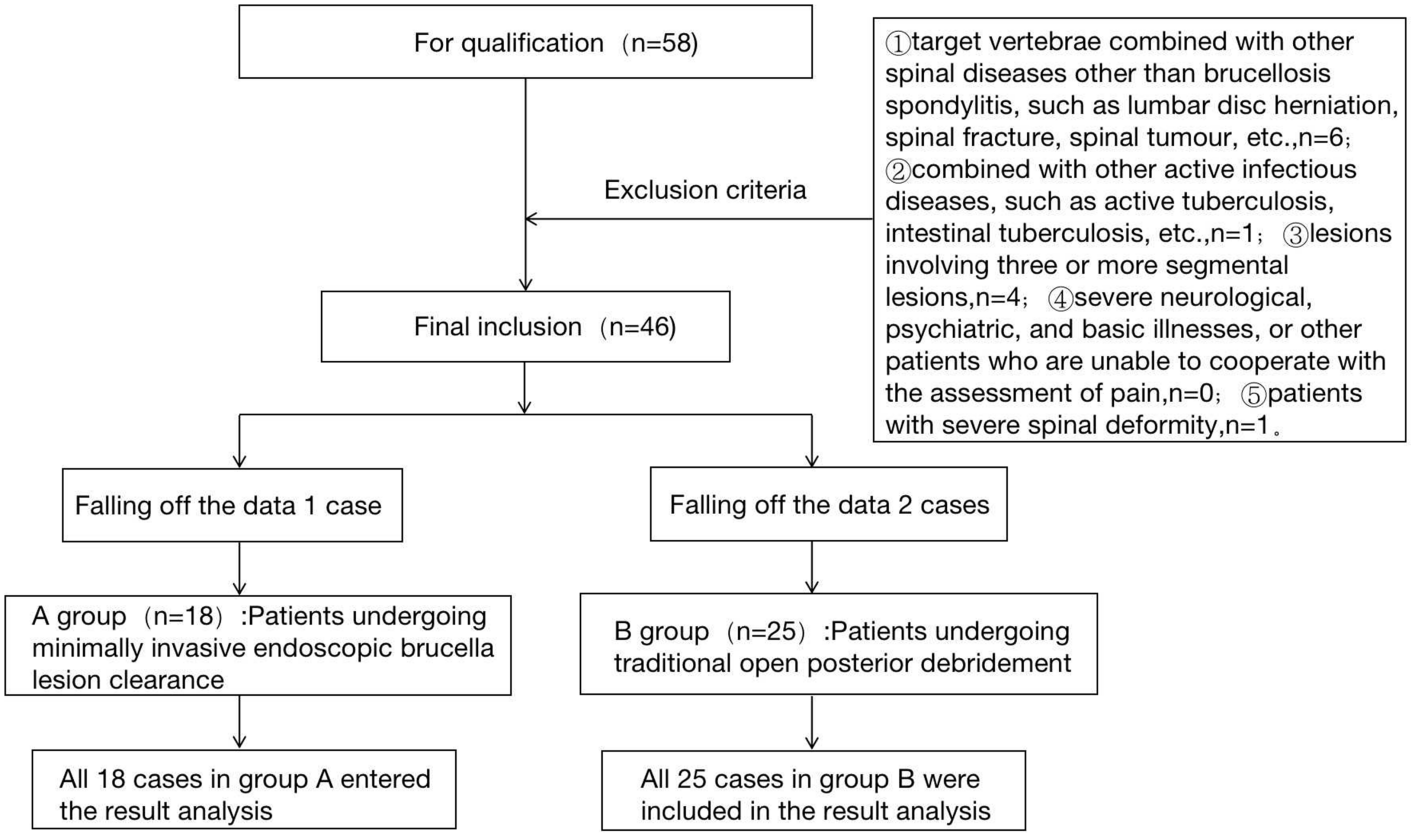
Flow chart of test grouping.

The flow chart of the two groups is shown in Figure 1.

### 3.3 Preoperative general data of patients in the two groups

Age, gender, body mass index, past history, ESR, CRP, PCT, D-dimer, Hb, VAS score and ODI score of patients in the two groups were not statistically different (*p* > 0.05). See Table 1.

**Table 1:** Basic preoperative data of the two groups.

Items	B Group (*n* = 25)	A Group (*n* = 18)	*x^2^/t*	*p*
Age (x¯ ± s)	51.24 ± 10.97	50.78 ± 10.14	−0.141	1.000
Sex(n, Male/Female)	12/13	9/9	0.017	1.000
BMI(kg/㎡, x¯±S)	24.38 ± 3.83	23.38 ± 2.36	−1.051	3.600
Cardiovascular and cerebrovascular(n)	16	8	1.623	2.436
Respiratory system(n)	4	2	0.208	7.776
Endocrine system(n)	13	7	0.723	4.740
Metabolic diseases(n)	3	4	0.003	1.000
ESR	52.24 ± 10.10	48.67 ± 7.75	−1.252	2.616
CRP	77.18 ± 16.08	80.58 ± 15.27	0.699	5.856
PCT	0.32 ± 0.07	0.29 ± 0.08	−1.250	2.616
D-Dimer (mg/L)	0.83 ± 0.55	1.08 ± 0.56	1.445	1.872
Hb	127.92 ± 6.73	125.78 ± 7.10	−1.006	3.840
Preoperative lumbar VAS score (score)	7.16 ± 0.98	7.28 ± 0.95	0.391	8.376
Preoperative lumbar ODI score (%)	75.28 ± 6.36	73.72 ± 5.08	−0.859	4.740

### 3.4 Comparison of postoperative VAS scores and ODI scores between the two groups

There was no significant difference in VAS score and ODI score between the two groups of patients at 1 month, 3 months after surgery and at the last follow-up (*p* > 0.05). However, the postoperative VAS scores and ODI scores of the two groups were significantly lower than those before the operation, and the difference was significantly significant (*p* < 0.05). These detailed findings are presented in Table 2.

**Table 2:** Postoperative VAS and ODI scores in both groups.

Items	Follow-up time	B group (*n* = 25)	A group (*n* = 18)	*t*	*p*
Low back VAS score (x¯ ± s, score)	1 month	3.44 ± 1.00*	3.22 ± 0.80*	−0.760	5.424
	3 month	2.20 ± 0.64*	2.00 ± 0.59*	−1.036	3.672
	6 month	1.92 ± 0.57*	1.44 ± 0.51*	−2.811	0.096
Low back ODI score (x¯ ± s, %)	1 month	37.52 ± 5.18*	34.50 ± 7.73*	−1.534	1.596
	3 month	23.40 ± 4.83*	25.06 ± 5.52*	1.043	3.636
	6 month	20.96 ± 4.89*	19.72 ± 3.86*	−0.891	4.536

*indicates that there is a statistical difference compared with that before the operation, *p* < 0.05.

### 3.5 Comparison of laboratory test indexes between the two groups of patients on the 1st postoperative day and at the end of the postoperative period

Immediately after surgery and at the end of the monitoring period, comprehensive assessments of all testing parameters were performed for both groups. The results indicated no significant disparities in the measured outcomes between the groups (*p* > 0.05), establishing equivalence in the postoperative progress and responses between the two surgical methods. Refer to Table 3.

**Table 3:** Index of laboratory tests 1 day and last postoperative time in the two groups.

Items	Follow-up time	B Group (*n* = 25)	A Group (*n* = 18)	*t*	*p*
1 day after surgery Inspection index	ESR	58.58 ± 11.66	56.42 ± 11.41	−0.604	6.588
	CRP	92.49 ± 15.89	86.57 ± 14.15	−1.259	2.580
	PCT	0.25 ± 0.07	0.26 ± 0.05	0.547	7.044
	D-Dimer	3.42 ± 1.77	4.73 ± 2.89	1.709	1.188
	Hb	105.12 ± 11.45	110.61 ± 10.63	1.597	1.416
The last time after surgery Inspection index	ESR	34.42 ± 7.31	30.98 ± 8.69	−1.406	2.004
	CRP	58.00 ± 14.36	61.73 ± 20.16	0.710	5.784
	PCT	0.24 ± 0.07	0.26 ± 0.05	0.876	4.632
	D-Dimer	2.57 ± 1.55	3.40 ± 2.00	1.518	1.644
	Hb	105.00 ± 14.58	113.22 ± 11.54	1.984	0.648

### 3.6 Analysis of postoperative complications in the two groups

Postoperative complications in Group B included: dural tears (5 cases, 20%), muscular venous thrombosis (5 cases, 20%), deep vein thrombosis (3 cases, 12%), surgical site infection (3 cases, 12%), cerebrospinal fluid leakage (2 cases, 8%), wound edge necrosis (2 cases, 8%), sinus tract formation (1 case, 4%), and disease recurrence (4 cases, 16%). In Group A, the complications were: dural tears (4 cases, 22.22%), muscular venous thrombosis (3 cases, 16.67%), deep vein thrombosis (1 case, 5.56%), cerebrospinal fluid leakage (4 cases, 22.22%), with no cases of surgical site infection, wound edge necrosis, or sinus tract formation, and only 1 recurrence case (5.56%).

Comparative analysis revealed no statistically significant differences between groups in rates of dural tears, muscular venous thrombosis, deep vein thrombosis, surgical site infection, cerebrospinal fluid leakage, wound edge necrosis, sinus tract formation (all *p* > 0.05). See Table 4.

**Table 4:** Comparison of postoperative complications between the two groups.

Group	Dural laceration	Muscular calf vein thrombosis	DVT	Incision infections	CSF leak	Skin necrosis	Sinus formation	Postoperative recurrent rate
B group (*n* = 25)	5(20)	5(20)	3(12)	3(12)	2(8)	2(8)	1(4)	4(16)
A group (*n* = 18)	4(22.22)	3(16.66)	1(5.55)	0(0)	4(22.22)	0(0)	0(0)	1(5.55)
*X^2^*	0.031	0.077	0.515	2.322	1.763	1.510	0.737	4.074
*p*	10.320	9.384	5.676	1.536	2.208	2.628	4.692	0.528

### 3.7 Comparison of operation time, intraoperative bleeding and postoperative hospital stay between the two groups

In the comparative analysis of surgical operation durations between the two patient groups, it was found that Group A, which underwent minimally invasive endoscopic procedures, recorded significantly shorter operation times, averaging 108.22 min (±17.70), as compared to Group B, which underwent traditional open surgery and recorded an average duration of 137.28 min (±24.31). This statistically significant difference (*p* < 0.05) underscores the efficiency and reduced surgical burden associated with the minimally invasive techniques employed in Group A. Furthermore, the analysis of intraoperative blood loss revealed a marked reduction in Group A, where the average volume was only 56.67 mL (±18.47), substantially less than the 216.28 mL (±37.67) observed in Group B. This difference was statistically significant (*p* < 0.05), suggesting that the minimally invasive approach not only shortens operation time but also minimizes surgical invasiveness and potential complications related to blood loss. Additionally, when examining postoperative hospital stays, Group A demonstrated a significantly faster recovery, with an average hospitalization duration of just 4.11 days (±0.83), in stark contrast to Group B’s average stay of 6.96 days (±1.06). This significant reduction in recovery time (*p* < 0.05) further highlights the benefits of minimally invasive surgical techniques, which contribute to quicker patient turnover and reduced hospital resource utilization. See Table 5.

**Table 5:** Comparison of operative time, intraoperative hemorrhage and postoperative hospital stay between the two groups of patients.

Items	B Group (*n* = 25)	A Group (*n* = 18)	*t*	*p*
Operation time/min	137.28 ± 24.31	108.22 ± 17.70	−4.308	**<0.012**
Intraoperative bleeding/ml	216.28 ± 37.67	56.67 ± 18.47	−16.560	**<0.012**
Postoperative hospital stay/d	6.96 ± 1.06	4.11 ± 0.83	−9.481	**<0.012**

Data in bold indicates statistical difference.

### 3.8 Typical cases

**Figure 2:**
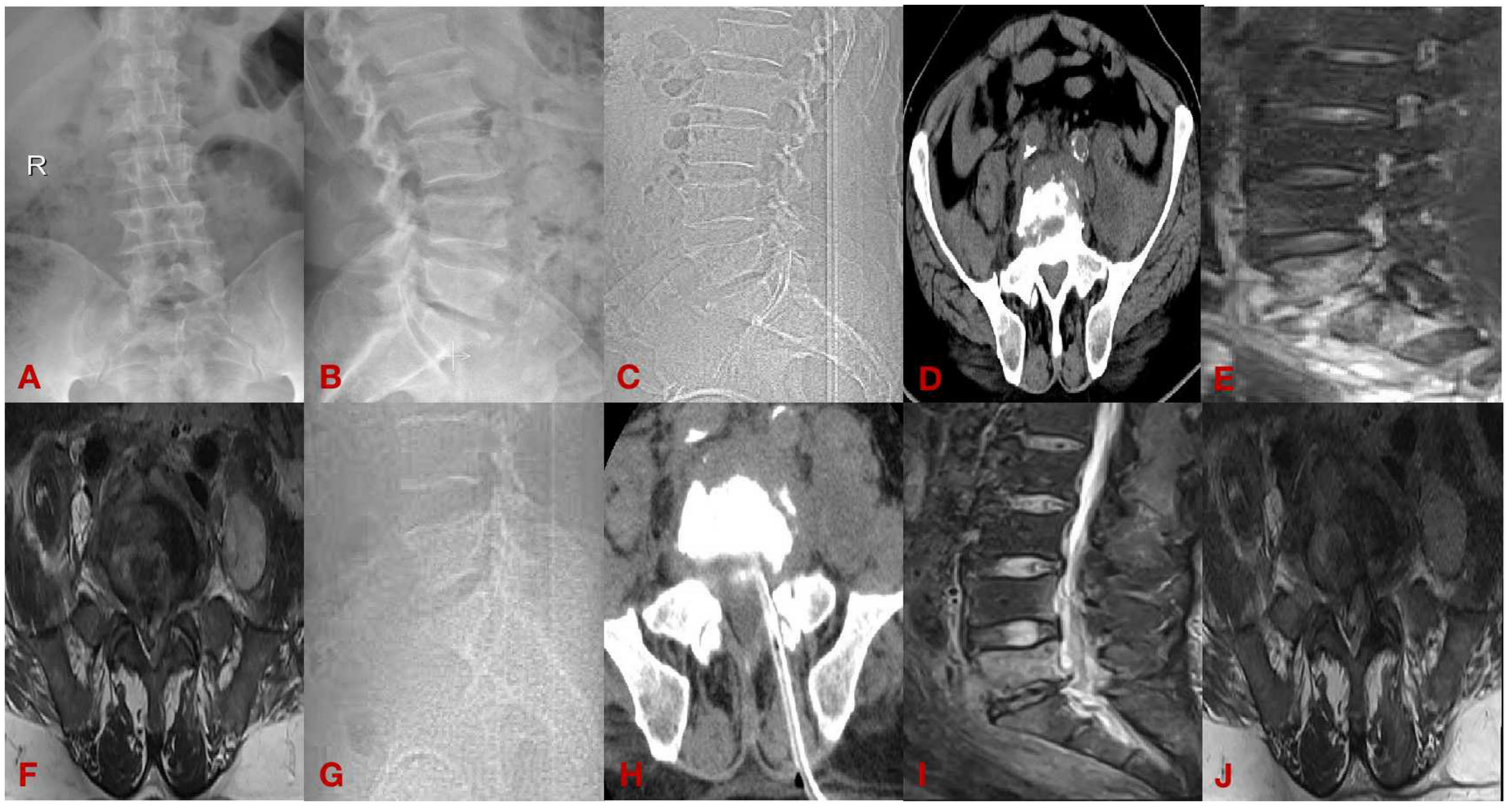
Case diagram of minimally invasive group. **(A,B)** Are preoperative DR; **(C,D)** are preoperative CT; **(E,F)** are preoperative MRI; **(G,H)** are postoperative CT; and **(I,J)** are postoperative MRI.

**Figure 3:**
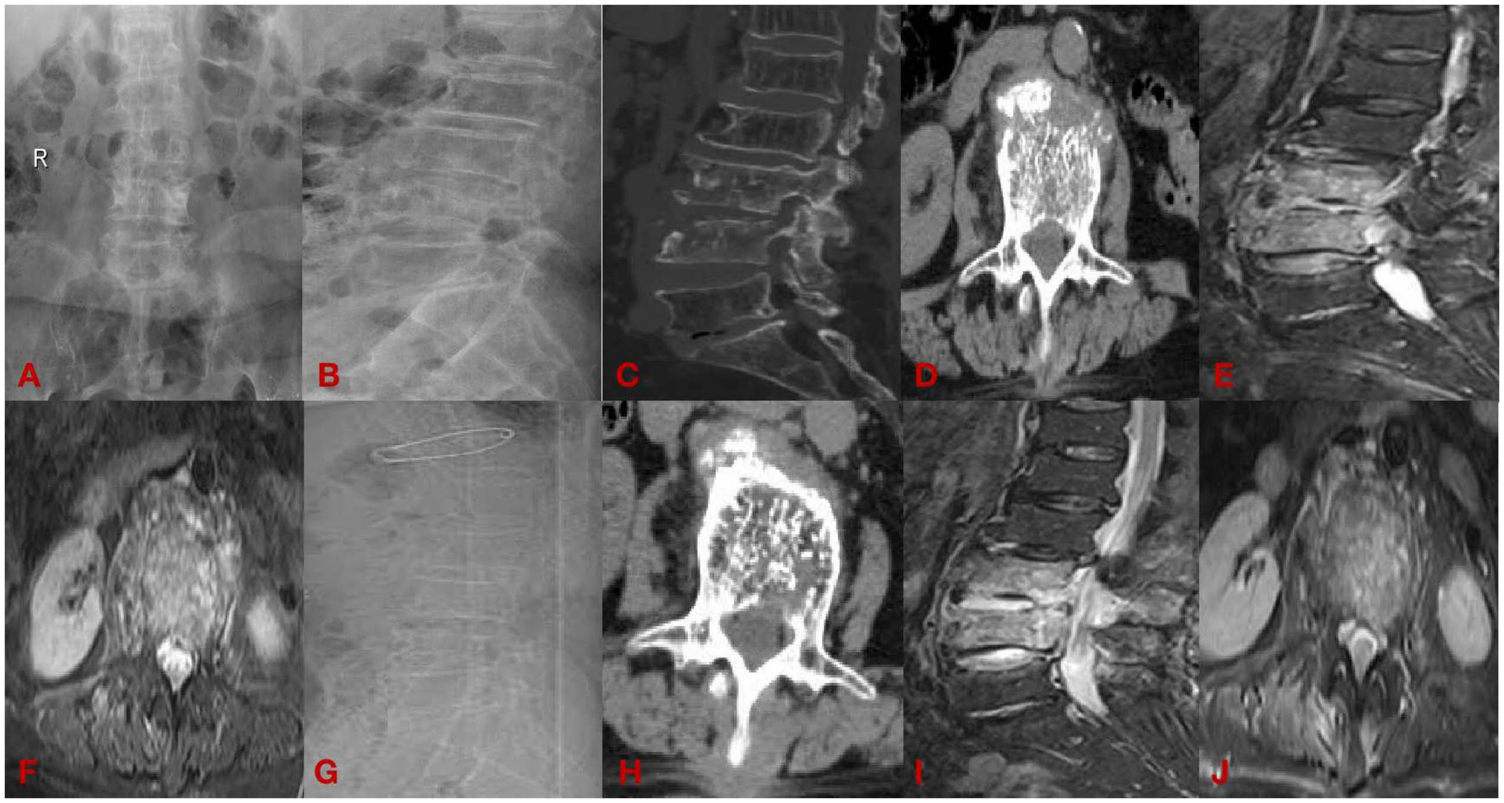
The open group case plot. **(A,B)** Are preoperative DR; **(C,D)** are preoperative CT; **(E,F)** are preoperative MRI; **(G,H)** are postoperative CT; and **(I,J)** are postoperative MRI.

(1) A 62-year-old male presenting with 8 months of lumbar spondylitis (L5-S1). See Figure 2.
(2) A 33-year-old man with chief complaint: low back pain with pain and discomfort of both lower limbs for 6 months for 1 week, diagnosed: brucella spondylitis (L3-4). See Figure 3.

## 4 Discussion

### 4.1 Summary of the evidence

Brucellar spondylitis, first clinically characterized by Kulowski and Vinke in 1932, represents a systemic infectious disease with distinctive pathological features including prolonged incubation periods and multi-organ involvement potential. Current epidemiological data indicate significant geographic variation in disease burden, with reported prevalence rates ranging from 6 to 58% across endemic regions (10). Patients with infection consistently exhibited comparable clinical symptoms, such as fever, diaphoresis, and localized pain (11, 12). The lumbar spine is most commonly affected, with subsequent involvement often seen in the thoracic and cervical regions of the spine (13–16). Despite the lack of precise epidemiological data in many endemic areas, it is estimated that globally, over 500,000 new cases are diagnosed each year (17). Traditionally, antimicrobial chemotherapy has been the cornerstone of treatment for Brucella spondylitis; however, it has shown limited efficacy in preventing the progression of kyphotic deformities and neurological deficits (18, 19). With the evolution of medical technology and a deeper understanding of the disease’s pathology, more aggressive surgical interventions have increasingly been recognized as preferable treatment options. Among these, the minimally invasive endoscopic removal of brucellosis lesions has emerged as a significant advancement. As medical technology has evolved and more is learned about the disease’s pathology, increasingly more aggressive surgical options have been accepted as preferable. Of these, the most significant improvements, have been the minimally invasive endoscopic removal of brucellosis lesions. The recent studies (20–22) have confirmed clinical effectiveness of this technique and further confirmed underlining good results.

Results of this study showed a considerable decrease of VAS and ODI scores after minimally invasive surgery compared to patients’ initial scores indicating significant subjective pain perception improvement. Wang (23) showed that patients with brucellosis spondylitis who had minimally invasive surgery reported a significant relief from both low back and radiating leg pain postoperatively. For low back and leg pain, the VAS, JOA, and ODI scores were also improved after the procedure, which continued to improve over time indicating the persistent advantages of minimally invasive approach. Chen (24) found surgery for brucellosis spondylitis improved VAS, neurological function, with no recurrences. Wang (25) concluded that patients who underwent minimally invasive lesion removal experienced significant improvements in both VAS and JOA scores 1 week after surgery, with further improvement observed by the time of the final follow-up. At the final follow-up visit, the JOA excellence rate reached 93%, demonstrating a high level of patient recovery. Additionally, Zhang (26) reported that the VAS and JOA scores 1 week postoperatively were significantly improved compared to preoperative levels, with sustained improvements at 1, 3, 6, and 12 months after surgery, all showing statistically significant differences. The results of this study are consistent with those of previous studies. These results corroborate the findings of previous studies, which collectively support the benefits of minimally invasive surgical techniques for brucellosis spondylitis patients in terms of pain reduction and functional recovery.

Traditional excision of spinal lesions through open surgery is known to be complex and result in a long recuperation time, and can cause significant medical trauma. It also has the risks of precipitating spinal instability as well as complications from the surgery itself like spinal cord and nerve root injury (27). Clearance of Brucella abscesses may result in dural tears and dural tears may contribute to complication of neurobrucellosis (28). Post-infectious complications typically exacerbate the patient’s clinical condition and significantly increase therapeutic complexity (29). Minimally invasive spinal approaches have recently found increased application in the management of spinal infection, representing a major shift in the management of spinal infections. These methods stress the importance of reduced surgical incisions, which governs less blood loss in operations, resulting in fewer postoperative complications, as well as more rapid return of the patients. As noted by Rajkumar (30), these surgical innovations extend particularly vital benefits to patients presenting with elevated health risks. Specifically, these techniques notably decrease the required duration for anesthesia, which directly enhances patient safety and comfort. Additionally, they allow for the implementation of sedation procedures as needed, effectively minimizing the overall trauma to the patient’s body. This approach not only conserves tissue integrity but also simplifies the complex process of wound healing, thereby improving the outcomes and quality of patient care. Zheng (20) showed that traditional open surgery required the removal of a large number of bony structures, which destabilized the spine, and was traumatic, bled a lot, and required long postoperative bed rest and a long recovery cycle, whereas endoscopy had shorter operative time, shorter bed rest, quicker pain relief, quicker postoperative recovery, and a shorter length of hospital stay. Liu (31) studied 18 patients with minimally invasive surgery The time spent was low, the blood loss was low, and all of them had no associated puncture tube placement complications, and the original tube placement wound healed well after extubation, with no sinus tract formation and no secondary infections. There were no intercostal nerve irritation symptoms, no spinal cord injury, and spinal cord compression symptoms did not become more severe. In a comprehensive retrospective study encompassing 128 patients diagnosed with spinal cord infections, Wang (32) demonstrated that the endoscopic excision of spinal lesions in the context of infection management provides a safety outcome comparable to that of traditional posterior surgeries. This method notably offers several advantages: it significantly reduces the duration of the operation, decreases the volume of blood lost during the procedure, and ensures a quicker recuperation with minimized postoperative drainage. These observations are corroborated by the results of the present study, which indicate that participants treated with minimally invasive surgical techniques benefited from markedly shorter surgical times, diminished intraoperative blood loss, reduced durations of postoperative hospitalization.

### 4.2 Limitations of the article

(1) This retrospective study has several inherent limitations, including a relatively small sample size, limited evaluation metrics, absence of power analysis, and potential selection bias.
(2) The application of minimally invasive approaches in brucellar spondylitis management remains poorly documented in the literature, necessitating additional clinical studies to establish their safety and efficacy profiles.

First, the relatively small sample size (*n* = 43) may limit statistical power, particularly for analyses of secondary outcomes, increasing the risk of failing to identify true differences. Second, as a single-center study, our results may be influenced by institution-specific clinical workflows and patient population characteristics, and thus require validation in different healthcare settings. Finally, while the non-randomized observational design reflects real-world clinical practice, it cannot fully control for confounding factors. Although we adjusted for them through multivariate regression, residual confounding may still exist.

These limitations suggest that the study results should be interpreted with caution, particularly the negative findings. Future research with larger sample sizes, multicenter prospective designs, and ideally randomized controlled trials is needed to further validate our observations.

## 5 Conclusion

The results of this study showed that minimally invasive endoscopic brucellosis lesion removal could achieve the same efficacy as compared with traditional open posterior lesion removal, but minimally invasive surgery has the advantages of shorter operative time, lower intraoperative hemorrhage and more obvious advantages in postoperative rehabilitation, etc., which makes it clinically feasible and effective procedure.

## Data Availability

The raw data supporting the conclusions of this article will be made available by the authors, without undue reservation.
